# FAIM2 Promotes Non-Small Cell Lung Cancer Cell Growth and Bone Metastasis by Activating the Wnt/β-Catenin Pathway

**DOI:** 10.3389/fonc.2021.690142

**Published:** 2021-09-09

**Authors:** Kelin She, Wensheng Yang, Mengna Li, Wei Xiong, Ming Zhou

**Affiliations:** ^1^National Health Commission (NHC) Key Laboratory of Carcinogenesis, Hunan Cancer Hospital and the Affiliated Cancer Hospital of Xiangya School of Medicine, Central South University, Changsha, China; ^2^Cancer Research Institute, Central South University, Changsha, China; ^3^Department of Thoracic Surgery, The Affiliated Shaoyang Hospital, Hengyang Medical School, University of South China, Shaoyang, China; ^4^The Key Laboratory of Carcinogenesis and Cancer Invasion of the Chinese Ministry of Education, Central South University, Changsha, China

**Keywords:** NSCLC, FAIM2, bone metastasis, Wnt pathway, EMT

## Abstract

**Aim:**

Bone metastasis is the major reason for the poor prognosis and high mortality rate of non-small cell lung cancer (NSCLC) patients. This study explored the function and underlying mechanism of Fas apoptotic inhibitory molecule 2 (FAIM2) in the bone metastasis of NSCLC.

**Methods:**

Samples of normal lung tissue and NSCLC tissue (with or without bone metastasis) were collected and analyzed for FAIM2 expression. HARA cells with FAIM2 overexpression and HARA-B4 cells with FAIM2 knockdown were tested for proliferation, migration, invasion, anoikis, and their ability to adhere to osteoblasts. Next, whether FAIM2 facilitates bone metastasis by regulating the epithelial mesenchymal transformation (EMT) process and Wnt/β-catenin signaling pathway were investigated. Finally, an *in vivo* model of NSCLC bone metastasis was established and used to further examine the influence of FAIM2 on bone metastasis.

**Results:**

FAIM2 was highly expressed in NSCLC tissues and NSCLC tissues with bone metastasis. FAIM2 expression was positively associated with the tumor stage, lymph node metastasis, bone metastasis, and poor prognosis of NSCLC. FAIM2 upregulation promoted HARA cell proliferation, migration, and invasion, but inhibited cell apoptosis. FAIM2 knockdown in HARA-B4 cells produced the opposite effects. HARA-B4 cells showed a stronger adhesive ability to osteocytes than did HARA cells. FAIM2 was found to be related to the adhesive ability of HARA and HARA-B4 cells to osteocytes. FAIM2 facilitated bone metastasis by regulating the EMT process and Wnt/β-catenin signaling pathway. Finally, FAIM2 was found to participate in regulating NSCLC bone metastasis *in vivo*.

**Conclusions:**

FAIM2 promoted NSCLC cell growth and bone metastasis by regulating the EMT process and Wnt/β-catenin signaling pathway. FAIM2 might be useful for diagnosing and treating NSCLC bone metastases.

## Introduction

Lung cancer is one of the most malignant cancers and accounts for nearly 12% of all human cancers worldwide, making it the leading cause of cancer-related death in both men and women (1). In general, lung cancer can be divided into categories of non-small cell lung cancer (NSCLC) and small cell lung cancer (SCLC), which account for 85% and 15% of all cases, respectively (2). Although targeted therapy and immune therapy have progressed in recent years due to the discovery of the epidermal growth factor receptor gene (*EGFR*), K-RAS, MET, and PD-l, the overall prognosis for lung cancer patients remains unsatisfactory (3, 4). Further studies of the underlying mechanism of lung cancer carcinogenesis and progression should provide novel options for treating lung cancer.

Distant metastasis is a major reason for the death of lung cancer patients. In NSCLC patients, bone tissue is a frequent site of metastasis and accounts for 13.2% of all metastasis sites (5). Although bone metastasis is a negative prognostic factor for NSCLC, the molecular mechanism of bone metastasis remains largely unknown (6, 7). Several studies have identified the molecules involved in modulating the bone metastasis of NSCLC. For instance, an upregulation of LIGHT/tumor necrosis factor superfamily member 14 (TNFSF14) signaling was found to result in the destruction of bone homeostasis in NSCLC patients and subsequent bone metastasis (8). Moreover, Wu et al. (9) reported that an ERBB2 mutation existed in 2% of NSCLC patients, and was responsible for bone metastasis. More investigations are needed to further explore the molecular mechanism of bone metastasis in NSCLC patients.

Tumor metastasis is a complicated process involving multiple steps, including epithelial mesenchymal transformation (10), effusion from blood vessels (11), formation of a pre-metastasis niche (12), and the colonization and proliferation of seeded cells (13). During the process of tumor metastasis, tumor cells fail to undergo apoptosis. Fas apoptotic inhibitory molecule 2 (FAIM2) is a member of the Fas apoptosis inhibitory molecule family, which can protect against the effects of Fas by direct interaction with the Fas receptor or regulate calcium release *via* interaction with Bcl-xL (14). Recent studies reported that FAIM2 participates in regulating tumor initiation and progression. For example, Wang et al. (15) discovered that microRNA-612 can modulate the tumorigenesis of neurofibromatosis type 1 by targeting FAIM2. Ziaee et al. (16) reported that FAIM2 expression was upregulated in colorectal adenocarcinoma tissues. With regard to lung cancer, Kang et al. (17) found that FAIM2 was highly expressed in SCLC tissue, and could serve as a novel diagnostic marker and potential therapeutic target for SCLC. Our previous studies revealed that FAIM2 was highly expressed in NSCLC tissues and played a role in modulating NSCLC cell proliferation, migration, invasion, and apoptosis (18, 19). However, until now, it has remained unclear whether FAIM2 participates in regulating NSCLC bone metastasis.

In this study, we investigated the expression profile of FAIM2 in NSCLC and examined its prognostic value, role in bone metastasis, and molecular mechanism. Our results suggest that FAIM2 could possibly serve as a biomarker and molecular target for treating NSCLC.

## Materials and Methods

### Clinical Specimens

Samples of NSCLC tissue and corresponding adjacent normal tissue (distance > 3 cm from the tumor tissue) were collected during the time period of September 2017 to October 2018. For patients without bone metastasis, the tissues were acquired during surgical resection. For patients with bone metastasis, the tissues were acquired during the pathological examination process (puncture biopsy). All diagnoses were independently confirmed by two pathologists. All tissue samples were obtained from patients who had not received any prior chemotherapy, radiotherapy, or other therapy, and had no other type of tumor. All patients provided their written informed consent for their participation in the study. Clinical information regarding the patients is shown in **Supplementary Material**. The protocols for all studies involving human participants were reviewed and approved by the Ethics Committee of the Affiliated Cancer Hospital of Xiangya School of Medicine.

### Cell Lines and Culture Procedure

Human NSCLC cell lines, including the lung squamous cell carcinoma HARA cell line and its bone seeking subclone HARA-B4, lung adenocarcinoma NCI-H1395 and A549 cells, embryonic fibroblast NIH3T3 cells, and primary osteoblasts (Primary OB) and osteoblasts (MC3T3E1) were all purchased from the National Infrastructure of Cell Line Resource (Beijing, China). All cells were subjected to the STR assay before being sent to the purchaser and were cultured in Dulbecco’s Modified Eagle Media (DMEM) medium containing penicillin (100 units/mL), streptomycin (100 μg/mL), and fetal bovine serum [FBS, 10% (v/v)] at 37°C in a humidified incubator containing 5% CO_2_. To inactivate the Wnt signaling pathway, 30 μmol/L of IWP-2 (Sigma-Aldrich, St. Louis, MO, USA) was added to the culture medium for 72 h.

### Quantitative Real-Time PCR

The tissues and cell were washed with PBS (pH 7.4), and the total RNA in tissues and cells was extracted using the Trizol reagent (Takara, Japan). cDNA was synthesized using a PrimeScript RT-PCR kit (Takara, Japan). Gene expression was detected by real-time PCR performed on a 7500 real-time PCR system (Applied Biosystems, Waltham, MA, USA). The 2^-△△CT^ method was used to calculate relative levels of gene expression, and GAPDH served as a housekeeping gene. The primers used for qRT-PCR are shown in **Table 1**.

**Table 1:** The information of the primers for real-time PCR.

Name	Sequence (5’-3’)	Length (bp)
FAIM2 F	CCAGGGAAAGCTCTCCGTG	203
FAIM2 R	GGTCCACATAGGCCCAGCTA	
ALP F	ACTGGGGCCTGAGATACCC	185
ALP R	TCGTGTTGCACTGGTTAAAGC	
RUNX2 F	TGGTTACTGTCATGGCGGGTA	101
RUNX2 R	TCTCAGATCGTTGAACCTTGCTA	
GAPDH F	TGTTCGTCATGGGTGTGAAC	154
GAPDH R	ATGGCATGGACTGTGGTCAT	

### Western Blotting

The total proteins in tissues and cells were extracted using the IP lysis buffer (Thermo Fisher) containing a proteinase and phosphatase inhibitor cocktail (Roche, Basel, Switzerland). The protein concentration in each extract was measured using a BCA protein assay kit (Thermo Fisher). Next, an aliquot of protein from each sample was diluted with a loading buffer and separated by sodium dodecyl sulfate polyacrylamide gel electrophoresis (SDS-PAGE). The protein bands were transferred onto a NC membrane (Merck, Kenilworth, NJ, USA), which was subsequently blocked with 5% fat-free milk at room temperature for 1 h. Next, the membrane was incubated with the primary antibody overnight at 4°C. The following antibodies against the proteins were used: FAIM2 (1:1,000, Abcam, Cat#: ab194435), ALP (1:1,000, Abcam, Cat#: ab154100), RUNX2 (1:1,000, Abcam, Cat#: ab23981), E-cadherin (1:1,000, Cell Signaling Technology, Cat#: #24E10), N-cadherin (1:1,000, Abcam, Cat#: ab18203), Vimentin (1:1,000, Abcam, Cat#: ab137321), and GAPDH (1:10,000, Abcam, Cat#: ab181602). The next day, the membrane was washed three times with TBST (pH 7.4) and then incubated with a secondary antibody (Thermo Fisher) at room temperature for 1 h. Finally, the protein bands were visualized and measured for staining intensity with an Odyssey imaging system (LI-COR Biosciences, Lincoln, NE, USA).

### Hematoxylin-Eosin Staining

Samples of normal lung tissue and lung cancer tissue were fixed in 10% paraformaldehyde solution. Following gradient dehydration and paraffin embedding, the tissues were cut into 6-μm thick sections, de-paraffinized in xylene solution, and then re-hydrated using a decreasing concentration gradient of ethanol solution. Next, the sections were stained with hematoxylin solution and eosin solution, respectively. After dehydration with ethanol solution and xylene solution, the staining results were observed under a microscope (Nikon, Japan).

### Immunohistochemistry Assay

IHC assays were carried out to test FAIM2 expression. Briefly, following the deparaffinization and hydration steps that were similar to those used in H&E staining, the sections were incubated with citrate antigen retrieval solution for 15 min and then with 0.2% Triton X-100 solution for 20 min. Internal peroxidase activity was quenched with 3% H_2_O_2_ solution. After being incubated with 5% normal goat serum to block non-specific binding, the sections were incubated overnight with the primary antibody against FAIM2 (1:2,000, Thermo Fisher Scientific, Cat#: PA520311). On the next day, the sections were incubated with the secondary antibody (1:4,000, Abcam, Cat#: ab205718) for 1 h. Finally, the sections were stained with hematoxylin solution and observed under a microscope.

### FAIM2 Overexpression, Knockdown, and Plasmid Transfection

To construct the FAIM2 overexpression plasmid, the full-length sequence of FAIM2 was inserted into the pcDNA3.0 plasmid, which was subsequently designated as pcD-FAIM2. Three shRNAs targeting FAIM2 (shFAIM2) were specifically designed to knock down FAIM2 expression in cells. Because HARA cells exhibited lower levels of FAIM2 expression, pcD-FAIM2 was transfected into HARA cells. shFAIM2 was transfected into HARA-B4 cells due to their higher levels of FAIM2 expression. Briefly, cells were cultured in 6-well plates until reaching 60% to 70% confluence; after which, they were transfected with pcD-FAIM2 or shFAIM2 by using Lipofectamine 2000 (Thermo Fisher, 11668027). pcDNA3.0 and shCTRL served as the negative controls. The transfection efficacies were evaluated *via* qRT-PCR and Western blotting.

### Cell Counting Kit-8 Assay

The viability of HARA and HARA-B4 cells was determined using the cell counting kit-8 (CCK-8) assay (Dojindo, Japan). Following stimulation, the cells were cultured in a 96-well plate (1 × 10^4^ cells per well) at 37°C in a 5% CO_2_ atmosphere for 0, 24, 48, or 72 h. Next, 10 μL of CCK-8 solution was added to each well and the cells were incubated for an additional 4 h at 37°C. Finally, the absorbance of each well at 450 nm was measured with a Microplate Reader (Bio-Tek Inc., Winooski, VT, USA).

### Colony Formation Assay

Following stimulation, cells were cultivated in a 6-well plate (1,000 cells per well) with the culture medium being replaced by fresh medium every 3 days for a period of 2 weeks. Next, the plate was washed with PBS and the cells were fixed with 1% paraformaldehyde solution for 20 min. A 0.1% crystal violet/40% methanol solution was used to stain the clones overnight. After removing the crystal violet and washing the culture plate with PBS, results of the colony formation assay were obtained using a scanner and subsequently analyzed using the Image J software.

### Edu Labeling and Immunofluorescence

Following stimulation, the cells were cultured on coverslips (Fisher, Pittsburgh, PA, USA) overnight at a density of 500 cells/mL; after which, they were incubated with Edu buffer for 1 h and then stained with anti-Edu antibody (Upstate, Temecula, CA, USA) according to the instructions provided by the manufacturer. DAPI (Merck, Germany) was used to stain the cell nucleus. Finally, images of the stained cells were obtained under a microscope (Nikon, Japan), and the percentages of EdU^+^ cells were calculated using the Image J software.

### *In Vitro* Osteogenic Differentiation

NIH3T3, primary OB, and MC3T3E1 cells were cultured in 6-well plates to 40%–50% confluence. Next, the culture medium was changed to α-MEM containing 10 nmol/L dexamethasone, 5 mmol/L glycerol phosphate, 100 units of penicillin, 100 μg/mL streptomycin, 0.25 μg/mL amphotericin B, 100 μmol/L L-ascorbic acid, and 10% FBS. To induce osteogenic differentiation, lipopolysaccharides (LPSs, 100 ng/mL) and a tumor growth factor beta 2 (TGF-β2) inhibitor were added to the culture medium. ALP activity was examined by using reagents in a QuantiChrom assay kit (BioAssay Systems, Hayward, CA, USA) after 7 days. Alizarin red solution was used for staining cells to visualize calcium accumulation. Finally, photographs were obtained under a microscope. A mixture of 20% methanol and 10% acetic acid was used to quantify Alizarin red S staining.

### Cell Adhesion Assay

HARA and HARA-B4 cells were transfected with GFP plasmid NIH3T3. Primary OB and MC3T3E1 cells were seeded into a 6-well plate and cultured overnight. Next, the HARA and HARA-B4 cells were added to the 6-well plate and co-cultured with the OB and MC3T3E1 cells for 24 h. Then, the cells were washed with PBS and fixed with 4% paraformaldehyde. Photographs were taken under a microscope and analyzed using the Image J software.

### Cell Anoikis Assay

To analyze HARA and HARA-B4 cell death due to a loss of adherence, the two cell types were stimulated and then seeded into low-adhesive plates at a density of 10 × 10^5^ cells per well. After 1, 4, or 7 days, the cells in each group were harvested and centrifuged at 1,000 rpm for 5 min; after which, they were washed with PBS, and then stained with Annexin V (Invitrogen, Carlsbad, CA, USA) solution and propidium iodide (PI) solution according to the instructions of the manufacturer. The percentage of cells with anoikis was analyzed by flow cytometry.

### Cell Apoptosis Assay

After receiving the appropriate stimulation, the cells in each group were harvested and centrifuged at 1,000 rpm for 5 min. The cells were then washed with PBS and stained with Annexin V (Invitrogen, USA) solution and propidium iodide (PI) solution according to the instructions of the manufacturer. The percentage of apoptotic cells was analyzed by flow cytometry.

### Two-Chamber Transwell Assay

The two-chamber Transwell assay was used to detect cell migration and invasion. After receiving the designated stimulation, HARA and HARA-B4 cells (2 × 10^4^ cells in 100 μL of FBS-free medium) were added to the upper chamber, and 700 μL of culture medium containing 10% FBS was added to the lower chamber. After being cultured at 37°C in a 5% CO_2_ atmosphere for 48 h, the non-migrated cells in the upper chamber were carefully removed, and the migrated cells on the Transwell membrane were washed with PBS, fixed with 4% paraformaldehyde, and stained using violet solution. Cell migration results were observed and photographed under a microscope (Nikon, Japan). The number of migrated cells in each field was counted.

Cell invasion was detected in a similar manner, except that the Transwell membrane was pre-coated with Matrigel (Corning, Corning, NY, USA).

### Cell Scratch Assay

After receiving the designated stimulation, 5 × 10^5^ HARA or HARA-B4 cells were cultured in a 6-well plate overnight. Next, a 200 μL pipette tip was used to remove cells and generate scratches on the cell monolayer. After rinsing with PBS to clear the redundant cells, the cells were cultured in FBS-free medium for 24 or 48 h. The scratches were then observed and photographed under a microscope (MOTIC, Xiamen, China). The data were analyzed using the IPP software.

### Immunofluorescence Staining

E-cadherin, N-cadherin, and Vimentin expression in cells was detected by immunofluorescence. Briefly, cells were seeded into immunofluorescence-specialized 12-well plates at a concentration of 1 × 10^5^ cells/well and cultured overnight. After being washed with PBS, the cells were fixed with 4% polyformaldehyde for 15 min, permeabilized with 0.1% TritonX-100 for 30 s, and then blocked with 10% BSA for 1 h. Next, the cells were washed three times with PBS and then incubated overnight with primary antibodies against E-cadherin (1:500, ab40772), N-cadherin (1:500, ab18203), and Vimentin (1:1,000, ab92547) at 4°C. On the next day, the cells were washed with PBS and then incubated with an Alexa Fluor 488 (1: 300, ab150077) secondary antibody for 60 min at room temperature. Finally, the cells were incubated with DAPI, covered with sealing liquid, and photographed under a microscope (Nikon, Japan).

### *In Vivo* Animal Experiments

Female BALB/c nude mice (4–5 weeks old) were housed in the SPF level barrier system at 26°C– 28°C with a free access to food and water. After the mice had been fed at our facility for 1 week, 1 × 10^6^ HARA cells transfected with pcDNA3.0 or pcD-FAIM2, as well as HARA-B4 cells transfected with shCTRL or shFAIM2, were injected into the outer tibia stick to observe the growth of tibia tumors in the hind limbs of the nude mice (20). A total of five mice were included in each group. After 5 weeks, the mice were sacrificed by the induction of an acute hemorrhage. The lung and tibia tissues of the mice were collected and stained with H&E to observe changes in the microstructure and test for FAIM2 expression. All experiments using mice were performed in accordance with guidelines in the *National Institute of Health’s Guide for the Care and Use of Laboratory Animals*, and the protocols were approved by the Ethics Committee of the Affiliated Cancer Hospital of Xiangya School of Medicine.

### Statistical Analysis

SPSS 13.0 software and GraphPad 7.0 software were used to perform all statistical analyses. *P*-values for differences between two groups were calculated using the two-tailed student’s t-test, and *P*-values for differences among three or more groups were determined by ANOVA. The Kaplan-Meier method was used to calculate cumulative survival curves, and the significance of a difference was assessed by the log-rank test. Chi-square values were calculated for correlation analyses. Data are presented as a mean value ± S.D. Statistical significance is indicated as follows: *P* > 0.05 = ns; *P* < 0.05 = *, ^#^; *P* < 0.01 = **, ^##^.

## Results

### FAIM2 Was Highly Expressed in NSCLC Tissues and Correlated With Bone Metastasis and a Poor Prognosis

**Figure 1:**
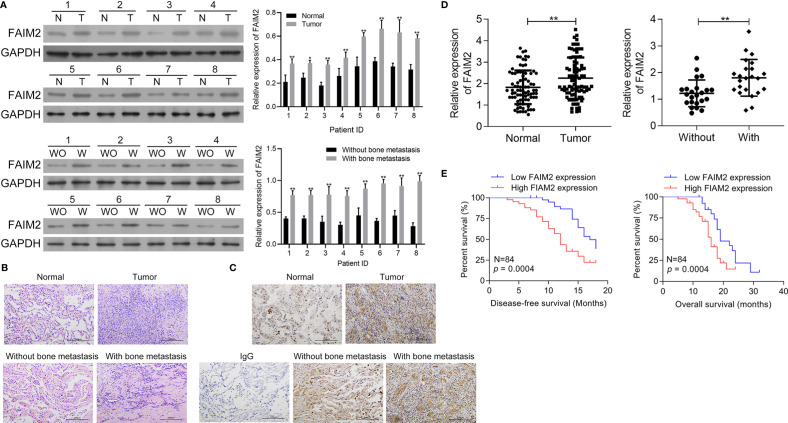
FAIM2 was highly expressed in NSCLC and correlated with bone metastasis and a poor prognosis. **(A)** The levels of FAIM2 protein expression in normal lung tissues and NSCLC tissues, as well as in NSCLC tissues with (W) or without (WO) bone metastasis, were examined by Western blotting. **(B)** The microstructure of normal lung tissues and NSCLC tissues, as well as NSCLC tissues with (W) or without (WO) bone metastasis. **(C, D)** FAIM2 protein and mRNA expression in normal lung tissues and NSCLC tissues, as well as in NSCLC tissues with (W) or without (WO) bone metastasis, were examined by IHC and qRT-PCR. **(E)** Kaplan-Meier curves for the disease-free survival (DFS) and overall survival (OS) of patients with high and low levels of FAIM2. *P* < 0.05, *; *P* < 0.01, ** *vs.* Normal lung tissues or NSCLC tissues without bone metastasis.

First, the levels of FAIM2 expression in NSCLC tumor tissues and adjacent normal tissues, as well as in NSCLC tissues with or without bone metastasis were examined by Western blotting. Those results showed that when compared to normal lung tissues, FAIM2 was more highly expressed in NSCLC tissues (*P* < 0.05 or *P* < 0.01, **Figure 1A**). Moreover, when compared to NSCLC tissues without bone metastasis, FAIM2 expression was upregulated in NSCLC tissues with bone metastasis (*P* < 0.01). H&E staining was performed to reveal the microstructure of normal lung tissues and NSCLC tissues, as well as NSCLC tissues with or without bone metastasis (**Figure 1B**). The normal lung tissues showed a normal microstructure with blue-purple nuclei and pink cytoplasm, while NSCLC tissues had an increased number of cells. Furthermore, relative to the NSCLS tissues without bone metastasis, the NSCLC tissues with bone metastasis displayed increased cell numbers and unclear cell boundaries. IHC assay results (**Figure 1C**) also showed that FAIM2 expression was increased in the NSCLC tissues relative to the normal lung tissues. When compared to the NSCLC tissues without bone metastasis, FAIM2 expression was increased in the NSCLC tissues with bone metastasis. Similar results were found for FAIM2 mRNA expression in the normal lung tissues and NSCLC tissues, as well as in the NSCLC tissues with or without bone metastasis (*P* < 0.01, **Figure 1D**). Moreover, we also analyzed the clinical significance of FAIM2 in NSCLC. Our results indicated that a high level of FAIM2 predicted a poor prognosis for NSCLC patients, including shorter disease-free survival (DFS) and overall survival (OS) times (**Figure 1E**). By exploring the association between FAIM2 expression and multiple clinicopathological characteristics of NSCLC patients, we discovered that FAIM2 was an independent factor correlated with the tumor stage (P = 0.0024), lymph node metastasis (*P* = 0.0161), and bone metastasis (*P* = 0.0010) (**Table 2**). The results above demonstrated that FAIM2 was highly expressed in NSCLC tissues and correlated with bone metastasis and a poor prognosis.

**Table 2:** Correlation between FAIM2 expression and multiple clinicopathological characteristics in NSCLC patients.

Clinicopathological characteristics		FIAM2 expression		Total	χ^2^	*p-*value
		Low	High			
Age	≤60	23	22	45	0.0478	0.8268
	>60	19	20	39		
Gender	Female	21	22	44	0.1112	0.7833
	Male	21	19	40		
Tumor stage	I	21	8	29	12.09	**0.0024**
	II	14	14	28		
	III	7	20	27		
Lymph node metastasis	Negative	25	14	39	5.791	**0.0161**
	Positive	17	28	45		
Bone metastasis	Negative	27	12	39	10.77	**0.0010**
	Positive	15	30	45		
T stage	T1	19	16	35	0.4431	0.8031
	T2	9	10	19		
	T3	14	16	30		
	Total			84		

Bold values means p < 0.05.

### FAIM2 Was Highly Expressed in NSCLC Cells and Associated With Cell Metastasis

**Figure 2:**
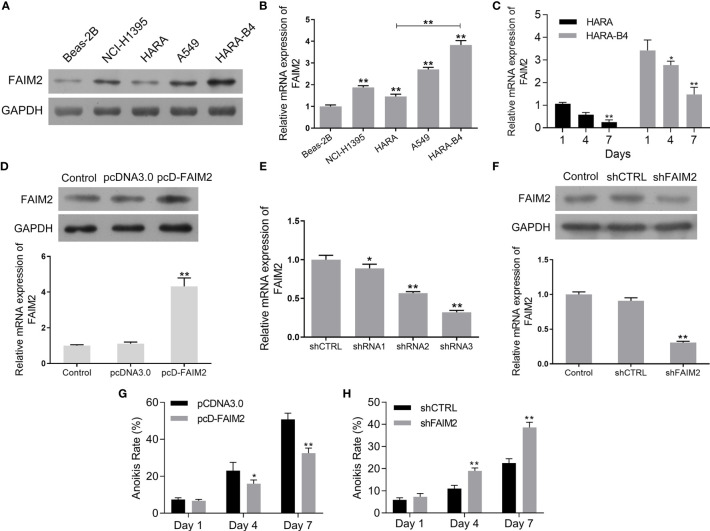
FAIM2 was highly expressed in NSCLC cells and related to cell metastasis. **(A, B)** FAIM2 protein and mRNA expression in Beas-2B, NCI-H1395, HARA, A549, and HARA-B4 cells was examined by Western blotting and qRT-PCR. **(C)** qRT-PCR was used to detect FAIM2 mRNA expression in HARA and HARA-B4 cells cultured under anchorage-dependent conditions during day 1 to day 7. **(D)** Following pcDNA3.0 or pcD-FAIM2 transfection, FAIM2 protein and mRNA expression in HARA cells was examined by Western blotting and qRT-PCR. **(E)** FAIM2 mRNA expression in HARA-B4 cells transfected with shRNA1, shRNA2, or shRNA3 was examined by qRT-PCR. **(F)** FAIM2 protein and mRNA expression in HARA cells transfected with shCTRL or sh-FAIM2 was examined by Western blotting and qRT-PCR. **(G)** Following transfection with pcDNA3.0 or pcD-FAIM2, HARA cell anoikis due to a loss of adherence for 1, 4, and 7 days was detected. **(H)** Following transfection with shCTRL or sh-FAIM2, HARA-B4 cell anoikis due to a loss of adherence for 1, 4, and 7 days was detected. *P* < 0.05, *; *P* < 0.01, ** *vs.* Beas-2B, Day1, the pcDNA3.0 or shCTRL group.

To further evaluate the function of FAIM2 in the bone metastasis of NSCLC, we compared the levels of FAIM2 expression in Beas-2B cells, NCI-H1395 cells, HARA cells, A549 cells, and HARA-B4 cells. Our results showed that when compared to Beas-2B cells, the levels of FAIM2 protein and mRNA expression in NSCLC cells were increased (**Figures 2A, B**, *P* < 0.01 for the mRNA level). Moreover, relative to HARA cells, the levels of FAIM2 were increased in HARA-B4 cells (*P* < 0.01 for the mRNA level), indicating a correlation between FAIM2 and bone metastasis. To analyze the relationship between anchorage-dependent cell growth and FAIM2 expression, the HARA and HARA-B4 cells were cultured in low-adhesive dishes and FAIM2 mRNA expression was measured. **Figure 2C** shows that FAIM2 expression in the HARA and HARA-B4 cells gradually decreased from day 1 to day 7, suggesting that FAIM2 is related to an anchorage-dependent cell growth. Next, HARA cells were transfected with pcD-FAIM2 to upregulate FAIM2 expression, while HARA-B4 cells were transfected with shFAIM2 to knockdown FAIM2 expression. Results in **Figure 2D** show that following pcD-FAIM2 transfection, the levels of FAIM2 protein and mRNA expression in HARA cells were increased (*P* < 0.01 for the mRNA level). Three shRNAs targeting FAIM2 were designed. ShRNA3 showed the best FAIM2 knockdown efficiency (**Figure 2E**), and was selected for use in subsequent experiments. Following shFAIM2 transfection, the levels FAIM2 protein and mRNA expression in HARA-B4 cells were both reduced (**Figure 2F**, *P* < 0.01 for the mRNA level). Moreover, anoikis was detected in HARA cells transfected with pcD-FAIM2 and in HARA-B4 cells transfected with shFAIM2. Transfection with pcD-FAIM2 reduced the anoikis of HARA cells on Day 4 (*P* < 0.05) and Day 7 (*P* < 0.01) (**Figure 2G**), while transfection with shFAIM2 increased the anoikis of HARA-B4 cells on Day 4 (*P* < 0.01) and Day 7 (*P* < 0.01) (**Figure 2H**). The results above demonstrated that FAIM2 was highly expressed in NSCLC cells and related to cell metastasis.

### Upregulation of FAIM2 Promoted HARA Cell Proliferation, Migration, and Invasion, but Reduced Cell Apoptosis

**Figure 3:**
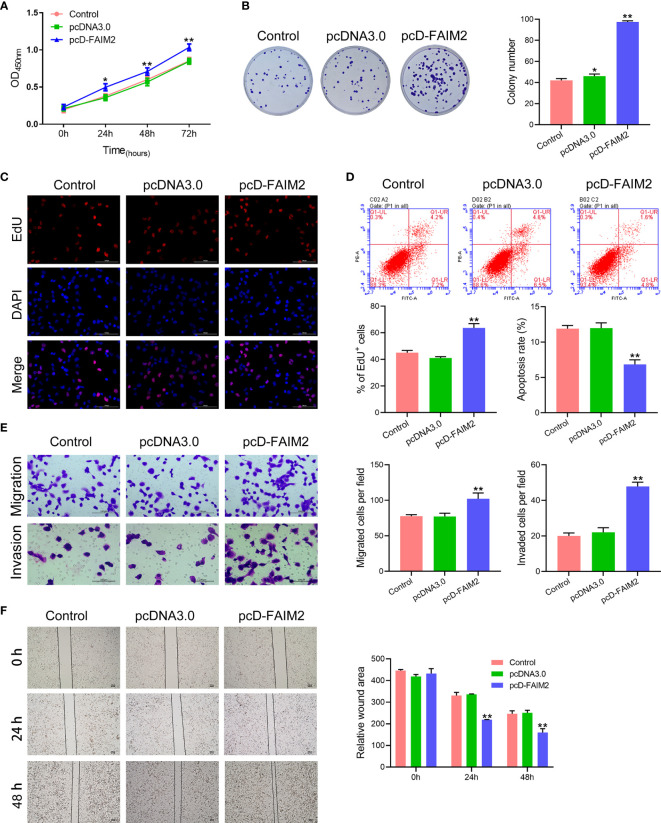
Upregulation of FAIM2 promoted HARA cell proliferation, migration, and invasion, but reduced cell apoptosis. Following pcDNA3.0 or pcD-FAIM2 transfection, **(A)** the viability of HARA cells was detected *via* the CCK-8 assay. **(B, C)** HARA cell proliferation was evaluated *via* the colony formation assay and EdU staining. **(D)** HARA cell apoptosis was assessed by flow cytometry. **(E)** HARA cell migration and invasion were measured *via* the two-chamber Transwell assay. **(F)** The cell scratch assay was conducted to further test for HARA cell migration. *P* < 0.05, *; *P* < 0.01, ** *vs.* the pcDNA3.0 group.

After being transfected with pcD-FAIM2, the viability, proliferation, migration, and invasion of HARA cells were detected. As shown in **Figure 3A**, pcD-FAIM2 transfection increased the OD_450_ values of HARA cells after 24, 48, and 72 h of culture, suggesting that pcD-FAIM2 promoted HARA cell viability. Furthermore, pcD-FAIM2 also promoted HARA cell proliferation, as evidenced by an increase in the colony numbers (*P* < 0.01) and percentage of EdU^+^ cells (*P* < 0.01, **Figures 3B, C**). Moreover, pcD-FAIM2 also inhibited the apoptosis of HARA cells (P < 0.01, **Figure 3D**). Both the numbers of migrated and invaded HARA cells in the pcD-FAIM2 group were notably increased (*P* < 0.01, **Figure 3E**), indicating that pcD-FAIM2 promoted HARA cell migration and invasion. The increased migration ability of the transfected cells was also demonstrated by the decreased relative wound areas in the pcD-FAIM2-transfected group at 24 and 48 h (*P* < 0.01, **Figure 3F**). The results above suggested that the overexpression of FAIM2 increased HARA cell proliferation, migration, and invasion, and inhibited cell apoptosis.

### Downregulation of FAIM2 Reduced HARA-B4 Cell Proliferation, Migration, and Invasion, but Promoted Cell Apoptosis

**Figure 4:**
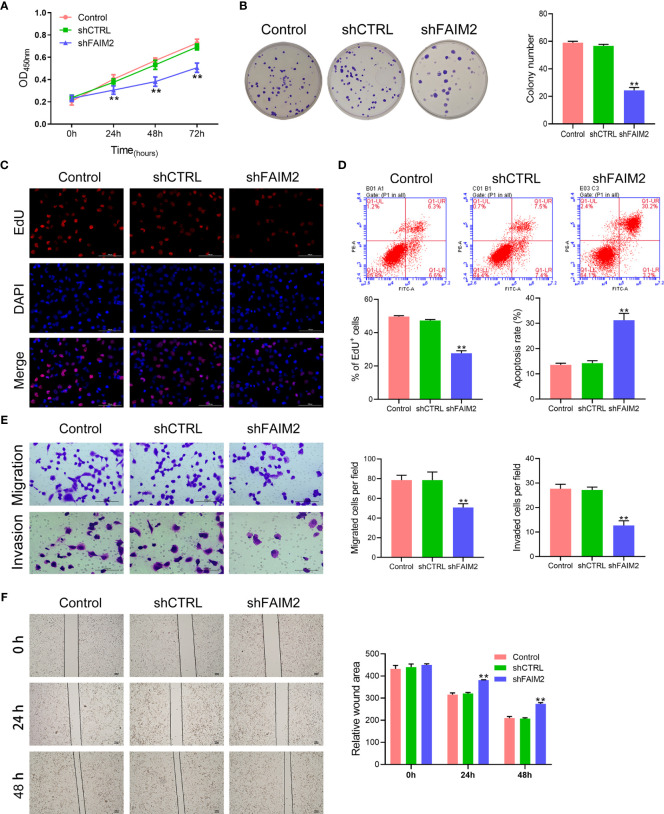
Downregulation of FAIM2 reduced HARA-B4 cell proliferation, migration, and invasion, but promoted cell apoptosis. Following shCTRL or shFAIM2 transfection, **(A)** the viability of HARA-B4 cells was detected *via* the CCK-8 assay. **(B, C)** HARA-B4 cell proliferation was evaluated *via* the colony formation assay and EdU staining. **(D)** HARA-B4 cell apoptosis was assessed by flow cytometry. **(E)** HARA-B4 cell migration and invasion were measured *via* the two-chamber Transwell assay. **(F)** The cell scratch assay was conducted to further test for HARA-B4 cell migration. *P* < 0.01, ** *vs.* the shCTRL group.

After shFAIM2 transfection, the viability, proliferation, migration, and invasion of HARA-B4 cells were detected. As shown in **Figure 4A**, shFAIM2 transfection lowered the OD_450_ nm values at 24, 48, and 72 h, suggesting that shFAIM2 reduced HARA-B4 cell viability. **Figures 4B, C** show that shFAIM2 inhibited HARA-B4 cell proliferation, as evidenced by the decreased colony numbers (*P* < 0.01) and percentages of EdU^+^ cells (*P* < 0.01). Moreover, shFAIM2 increased the apoptosis of HARA-B4 cells (*P* < 0.01, **Figure 4D**), but decreased the numbers of migrated and invaded HARA-B4 cells (*P* < 0.01, **Figure 4E**), indicating that shFAIM2 inhibited HARA-B4 cell migration and invasion. Finally, shFAIM2 reduced HARA-B4 cell migration, as evidenced by the increased relative wound areas in the shFAIM2 group at 24 and 48 h (*P* < 0.01, **Figure 4F**). These results showed that downregulation of FAIM2 reduced HARA-B4 cell proliferation, migration, and invasion, and promoted cell apoptosis.

### Primary OB and MC3T3E1 Cells Were Effective for Inducing Osteoblast Differentiation

Embryonic fibroblast NIH3T3, primary OB, and osteoblast MC3T3E1 cells were used to induce osteoblast differentiation. Following the induction of osteoblast differentiation, the levels of ALP and RUNX2 protein and mRNA in the cells were measured. Results showed that after the induction of osteoblast differentiation, the levels of both ALP and RUNX2 expressions in NIH3T3, Primary OB and MC3T3E1 cells were upregulated (*P* < 0.05 or *P* < 0.01 for the mRNA level) (**Supplementary Figures 1A, B**). ALP staining and Alizarin Red staining were performed to further verify the successful establishment of an osteoblast differentiation model. Those staining results showed that primary OB and MC3T3E1 cells induced a more intense staining than did NIH3t3 cells (*P* < 0.01, **Supplementary Figure 1C**). These results suggested that the primary OB and MC3T3E1 were effective for inducing osteoblast differentiation.

### HARA-B4 Cells Showed a Stronger Adhesive Ability to Osteocytes Than Did HARA Cells

Because HARA-B4 is a bone-seeking cell line, it was assumed that those cells would show a stronger adhesive ability to osteocytes. To verify this assumption, HARA and HARA-B4 cells were transfected with a plasmid containing GFP to detect the luciferase signaling. Next, following the co-culture of HARA (or HARA-B4) cells and NIH3T3 cells (or primary OB and MC3T3E1, cells that were subjected to the induction of osteoblast differentiation), the adhesive abilities of HARA-B4 and HARA cells were detected. Those results showed that HARA-B4 cells had a stronger ability to bind to osteocytes than did HARA cells (**Supplementary Figures 2A–C**, *P* < 0.01 in the MC3T3E1 and Primary OB cell groups).

### FAIM2 Was Related to the Ability of HARA and HARA-B4 Cells to Adhere to Osteocytes

**Figure 5:**
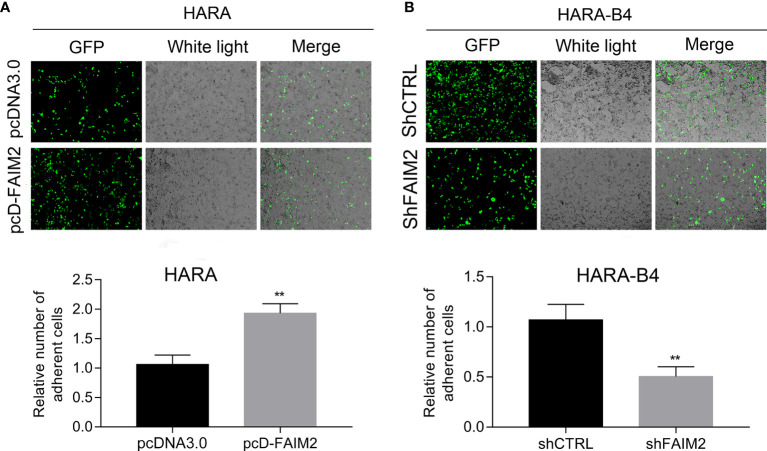
FAIM2 was related to the ability of HARA and HARA-B4 cells to adhere to osteocytes. **(A)** Following pcDNA3.0 or pcD-FAIM2 transfection, the ability of HARA cells to adhere to MC3T3E1 cells (cells were subjected to induction of osteoblast differentiation) was evaluated. **(B)** Following shCTRL or shFAIM2 transfection, the ability of HARA-B4 cells to adhere to MC3T3E1 cells (cells were subjected to induction of osteoblast differentiation) was evaluated. *P* < 0.01, ** *vs.* the pcDNA3.0 or shCTRL group.

Next, we assessed how FAIM2 modulated the adhesive ability of HARA and HARA-B4 cells to osteocytes. Following the co-culture with MC3T3E1 (cells were subjected to an induction of osteoblast differentiation), the relative numbers of adherent HARA and HARA-B4 cells were measured. Those measurements showed that an upregulation of FAIM2 expression in HARA cells significantly increased the adhesion of HARA cells to osteocytes (*P* < 0.01, **Figure 5A**), and a downregulation of FAIM2 expression in HARA-B4 cells reduced the adhesion of HARA-B4 cells to osteocytes (*P* < 0.01, **Figure 5B**). Those results suggested that FAIM2 could modulate the adhesive ability of NSCLC cells, and plays a role in promoting bone metastasis.

### FAIM2 Facilitated Bone Metastasis by Regulating the Epithelial Mesenchymal Transformation

**Figure 6:**
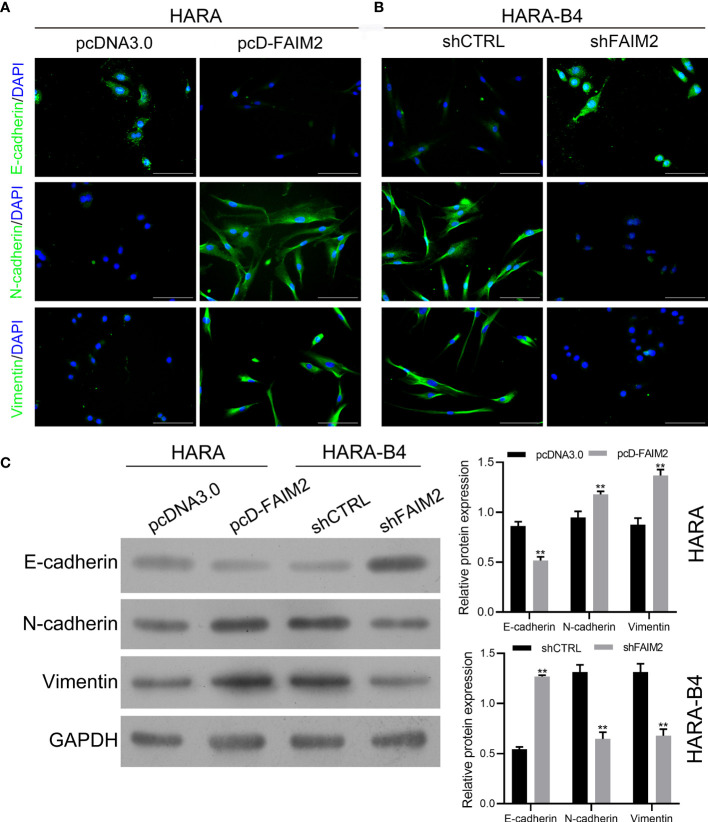
FAIM2 facilitated bone metastasis by regulating the EMT processes. Following pcDNA3.0 or pcD-FAIM2 transfection of HARA cells, as well as shCTRL or shFAIM2 transfection of HARA-B4 cells, **(A, B)** the expression of EMT markers, including E-cadherin, N-cadherin, and Vimentin was examined by immunofluorescence staining. **(C)** The levels of E-cadherin, N-cadherin, and Vimentin protein expression were determined by Western blotting. *P* < 0.01, ** *vs.* the pcDNA3.0 group.

To explore whether FAIM2 plays a role in regulating the EMT process in HARA and HARA-B4 cells, we detected the levels of E-cadherin, N-cadherin, and Vimentin expression in pcD-FAIM2-transfected HARA cells and shFAIM2-transfected HARA-B4 cells. IF assay results (**Figure 6A**) showed that pcD-FAIM2 transfection reduced E-cadherin expression, but increased N-cadherin and Vimentin expression in HARA cells, indicating that pcD-FAIM2 promoted the EMT process in HARA cells. When compared to HARA cells in the pcDNA3.0 group, HARA cells in the pcD-FAIM2 group displayed a spindle-shaped morphology. Moreover, shFAIM2 transfection enhanced E-cadherin expression, but decreased the levels of N-cadherin and Vimentin expression in HARA-B4 cells (**Figure 6B**), indicating that shFAIM2 attenuated the EMT process in HARAB4 cells. Similar results were shown by Western blotting (*P* < 0.01, **Figure 6C**). These results suggested that FAIM2 participates in modulating the EMT process in NSCLC cells, and can thereby promote bone metastasis.

### FAIM2 Promoted HARA Cell Migration and Invasion by Activating the Wnt Signaling Pathway

**Figure 7:**
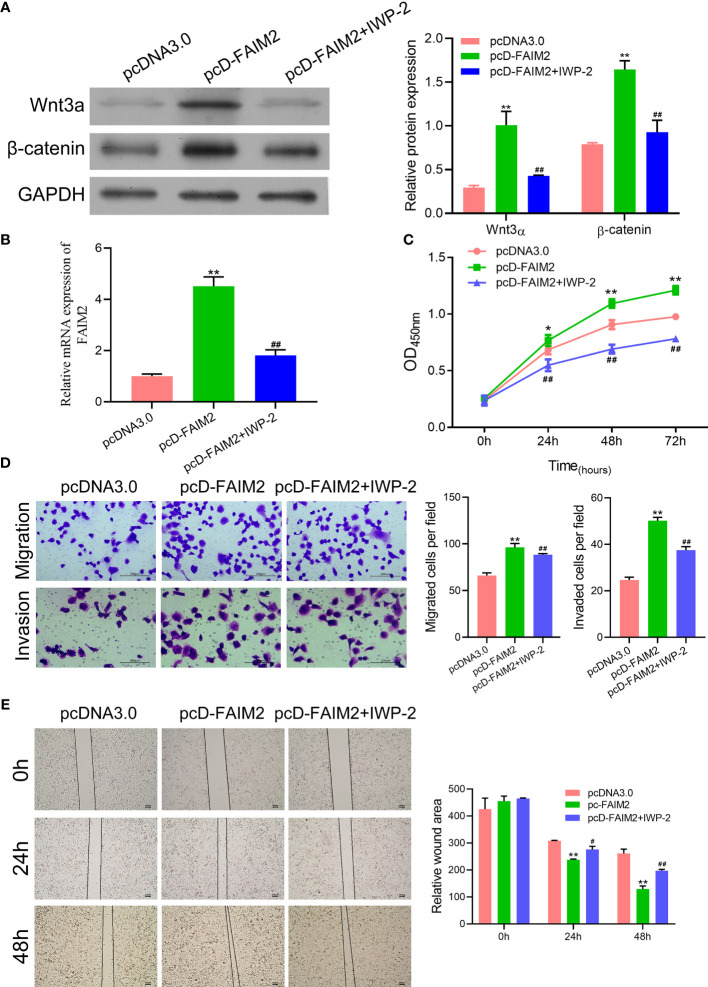
FAIM2 promoted HARA cell migration and invasion by activating the Wnt signaling pathway. Following pcD-FAIM2 transfection and IWP-2 treatment, **(A)** the levels of Wnt3a and β-catenin protein expression were examined by Western blotting. **(B)** FAIM2 mRNA expression was measured by qRT-PCR. **(C)** Cell viability was detected by the CCK-8 assay. **(D)** Cell migration and invasion were evaluated *via* the two-chamber Transwell assay. **(E)** The cell scratch assay was conducted to further test for HARA cell migration. *P* < 0.05, *; *P* < 0.01, ** *vs.* the pcDNA3.0 group; *P* < 0.05, ^#^; *P* < 0.01, ^##^ *vs.* the pcD-FAIM2 group.

Previous reports suggested that the Wnt signaling pathway is associated with the bone metastasis of NSCLC (21). Therefore, we examined whether FAIM2 might regulate HARA cell migration and invasion by modulating the Wnt signal pathway. We found that pcD-FAIM2 activated the Wnt pathway in HARA cells, as evidenced by the increased levels of Wnt3a and β-catenin protein and mRNA expression (*P* < 0.01, **Figure 7**). Next, IWP-2 was used to inactivate the Wnt pathway. Results showed that IWP-2 treatment significantly attenuated the Wnt pathway activation induced by pcD-FAIM2 (*P* < 0.01). When compared to the pcD-FAIM2 group, the levels of FAIM2 mRNA in HARA cells were significantly lower in the pcD-FAIM2+IWP-2 group (*P* < 0.01, **Figure 7B**). Furthermore, IWP-2 significantly reversed the pcD-FAIM2-induced increase in HARA cell viability (*P* < 0.01, **Figure 7C**). Moreover, when compared to the pcD-FAIM2 group, the numbers of migrated and invaded HARA cells in the pcD-FAIM2+IWP-2 group were notably decreased (*P* < 0.01, **Figure 7D**), indicating that IWP-2 also attenuated the migration and invasion of HARA cells induced by pcD-FAIM2. In addition, IWP-2 attenuated HARA cell migration, as evidenced by the increased relative wound areas in the pcD-FAIM2+IWP-2 group at 24 and 48 h (*P* < 0.05 or *P* < 0.01, **Figure 7E**). These results suggested that FAIM2 promoted HARA cell migration and invasion by activating the Wnt signaling pathway.

### FAIM2 Participated in Regulating NSCLC Bone Metastasis *In Vivo*

**Figure 8:**
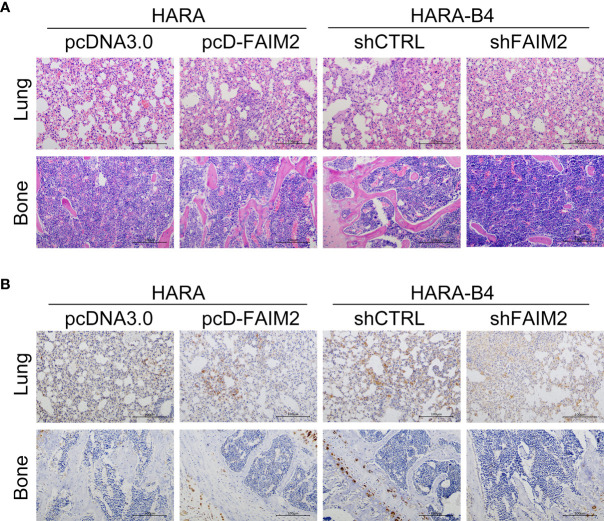
FAIM2 participated in regulating NSCLC bone metastasis *in vivo*. An *in vivo* NSCLC bone metastasis model was established. **(A)** The lung and bone tissues were collected and stained with H&E to observe changes in the microstructure. **(B)** The lung and bone tissues were collected to test for FAIM2 expression *via* IHC.

Finally, we investigated whether FAIM2 participates in modulating NSCLC bone metastasis *in vivo*. As shown in **Figure 8A**, the rate of HARA cell metastasis to bone tissue was higher in the pcD-FAIM2 group than in the pcDNA3.0 group, while the rate of HARA-B4 cell metastasis in the shFAIM2 group was lower than that in the shCTRL group. Furthermore, a comparison of bone tumor tissue microstructure showed that the blood supply available to tumors in the pcD-FAIM2 group was greater than that in the shFAIM2 group. IHC assays were performed to test for FAIM2 expression. Those results showed that the levels of FAIM2 expression in lung tissue and bone metastasis NSCLC tissues were higher in the pcD-FAIM2 group than in the shFAIM2 group (**Figure 8B**). Those results further suggest that FAIM2 participates in regulating NSCLC bone metastasis.

## Discussion

Tumor metastasis is the main reason for the poor prognosis of NSCLC patients (22). Because bone is the major metastasis site of NSCLC, inhibition of NSCLC bone metastasis is considered to be helpful for increasing the survival rate of NSCLC patients (7). FAIM2 belongs to the Fas apoptosis inhibitory molecule family (14). In the current study, we comprehensively investigated the expression pattern of FAIM2 in NSCLC. Moreover, we explored the influence of FAIM2 on the bone metastasis of NSCLC. In summary, our main findings were as follows: 1) FAIM2 was overexpressed in NSCLC tissues and NSCLC tissues with bone metastasis, and a high level of FAIM2 expression was correlated with a poor clinical prognosis; 2) FAIM2 was highly expressed in NSCLC cells and related to cell metastasis; 3) an upregulation of FAIM2 promoted HARA cell proliferation, migration, and invasion, but reduced cell apoptosis; 4) a downregulation of FAIM2 impaired HARA-B4 cell proliferation, migration, and invasion, but promoted cell apoptosis; 5) HARA-B4 cells showed a stronger adhesive ability than HARA cells, and FAIM2 promoted the adhesive ability of HARA-B4 cells to osteocytes; 6) FAIM2 promoted NSCLC bone metastasis by regulating the EMT process and activating the Wnt signaling pathway. Based on the results above, we deduced that FAIM2 might be a promising biomarker and target for inhibiting or treating bone metastasis in NSCLC patients.

FAIM2 is an anti-apoptotic protein that is upregulated in B cells, leading to apoptotic resistance and Fas-mediated cell death. In addition to their anti-apoptosis effect, FAIM family members have been discovered to participate in several physiological processes and pathological conditions, including cell proliferation, metabolic regulation, tumorigenesis, and Alzheimer’s disease (23). With regard to carcinogenesis, FAIM family members have been reported to be upregulated in multiple myeloma, which could promote cancer cell proliferation by activating the IGF-1 and AKT signaling pathways (24).

In recent years, numerous reports have provided evidence supporting the oncogenic role of FAIM2. Pan et al. (25) reported that de-differentiated OS cells with upregulated FAIM2 levels could acquire a survival ability that enhances their metastatic potential. Moreover, Chen et al. (26) reported that the LncRNA DCST1-AS1/miR-1254/FAIM2 axis facilitated tumor progression in hepatocellular carcinoma. In lung cancer, the upregulation of lncRNA-SNHG7 was reported to accelerate the proliferation, migration, and invasion of lung cancer cells by upregulating FAIM2 expression (18, 19). However, those previous studies focused on the upstream regulation of FAIM2, and ignored the downstream effectors of FAIM2 in tumor cells. In this study, we found that FAIM2 regulated the EMT process and Wnt pathway to promote tumor proliferation and metastasis. These results provide a useful supplement to former studies.

Bone metastasis is a comprehensive process which includes the development of osteogenic/osteolytic bone lesions (27). The Wnt signaling pathway plays an important role in bone development under physiological conditions, but is also deeply involved in tumor initiation and progression (28). EMT is an important process that tumor cells must complete to obtain their metastatic potential; as it enables them to detach from the original tumor, enter the circulation, and disseminate to the bone (28). Multiple studies have proven that the Wnt signaling pathway promotes the EMT process (29). For example, the activation of Wnt receptors FZD8 and LRP5 was reported to facilitate cell migration and invasion by accelerating EMT in prostate cancer (30, 31). Moreover, an upregulation of APC and β-catenin was shown to significantly increase the levels of matrix MMP-9 and MMP-2 (members of the metalloproteinase family) expression and downregulate E-cadherin, Snail1, and Zeb1 expression, which ultimately resulted in EMT and bone metastasis in lung cancer (32, 33).

Several unanswered questions remain to be explored in future studies. For example, the direct effector that was regulated by FAIM2 in the Wnt pathway remains unknown. Also, a quantitative evaluation of possible diagnostic tools and therapeutic agents based on FAIM2 must still be conducted.

In conclusion, our research showed that FAIM2 was significantly upregulated in NSCLC tissues and performed an oncogenic function in tumor progression, including tumor cell proliferation and bone metastasis. With regard to the molecular mechanism, we found that an upregulation of FAIM2 expression activated the EMT process and Wnt signaling pathway. FAIM2 also exhibited a diagnostic value in tumor progression. We found that FAIM2 expression was independently correlated with a poor survival and disease outcomes of NSCLC patients. Taken together, these results suggest FAIM2 as a potential diagnostic biomarker and therapeutic target for NSCLC patients.

## Data Availability Statement

The raw data supporting the conclusions of this article will be made available by the authors, without undue reservation.

## Ethics Statement

The studies involving human participants were reviewed and approved by the Ethics Committee of the Affiliated Cancer Hospital of Xiangya School of Medicine. The patients/participants provided their written informed consent to participate in this study.

## Author Contributions

MZ and KS conceived and designed the study, and provided administrative support. KS, WY, and ML performed the experiments. WX and MZ analyzed and interpreted the data. KS and WY wrote the manuscript. All authors contributed to the article and approved the submitted version.

## Funding

The study was supported by the Key Scientific and Technological project of Shaoyang City (2020NS36).

## Conflict of Interest

The authors declare that the research was conducted in the absence of any commercial or financial relationships that could be construed as a potential conflict of interest.

## Publisher’s Note

All claims expressed in this article are solely those of the authors and do not necessarily represent those of their affiliated organizations, or those of the publisher, the editors and the reviewers. Any product that may be evaluated in this article, or claim that may be made by its manufacturer, is not guaranteed or endorsed by the publisher.

## Supplementary Material

Supplementary Figure 1 Primary OB and MC3T3E1 cells were effective for inducing osteoblast differentiation. Following the induction of osteoblast differentiation, **(A)** the levels of ALP and RUNX2 protein and mRNA expression in cells were measured by qRT-PCR and western blotting. **(B)** ALP staining and Alizarin Red staining were performed to evaluate osteoblast formation. *P* * 0.05, *; *P* < 0.01, ** *vs.* Control group.

Supplementary Figure 2 HARA-B4 cells showed a stronger adhesive ability to osteocytes than did HARA cells HARA and HARA-B4 cells that had been transfected with the GFP plasmid were co-cultured with NIH3T3, Primary OB or MC3T3E1 cells. **(A–C)** The adhesive ability of HARA and HARA-B4 cells was measured. NS: not significant. *P* < 0.01, ** *vs.* HARA cells.
