# Supplementary figures and images for: FAIM2 Promotes Non-Small Cell Lung Cancer Cell Growth and Bone Metastasis by Activating the Wnt/β-Catenin Pathway
Source: Front Oncol. 2021 Sep 9;11:690142. doi: 10.3389/fonc.2021.690142 (PMC8459617; doi:10.3389/fonc.2021.690142)

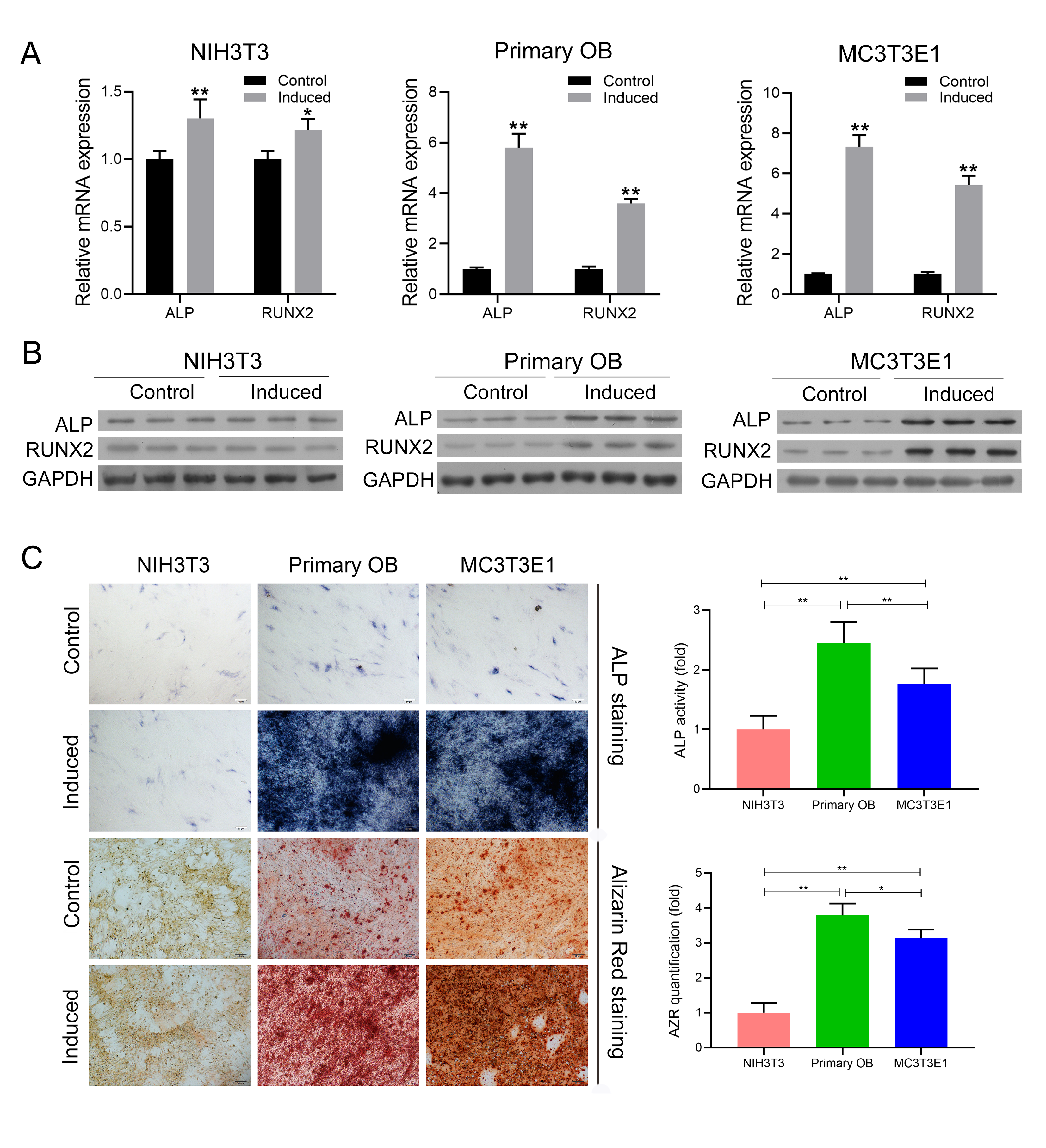

Supplement: Supplementary Figure 1 — Primary OB and MC3T3E1 cells were effective for inducing osteoblast differentiation. Following the induction of osteoblast differentiation, (A) the levels of ALP and RUNX2 protein and mRNA expression in cells were measured by qRT-PCR and western blotting. (B) ALP staining and Alizarin Red staining were performed to evaluate osteoblast formation. P * 0.05, *; P < 0.01, ** vs. Control group. [file Image_1.tif]

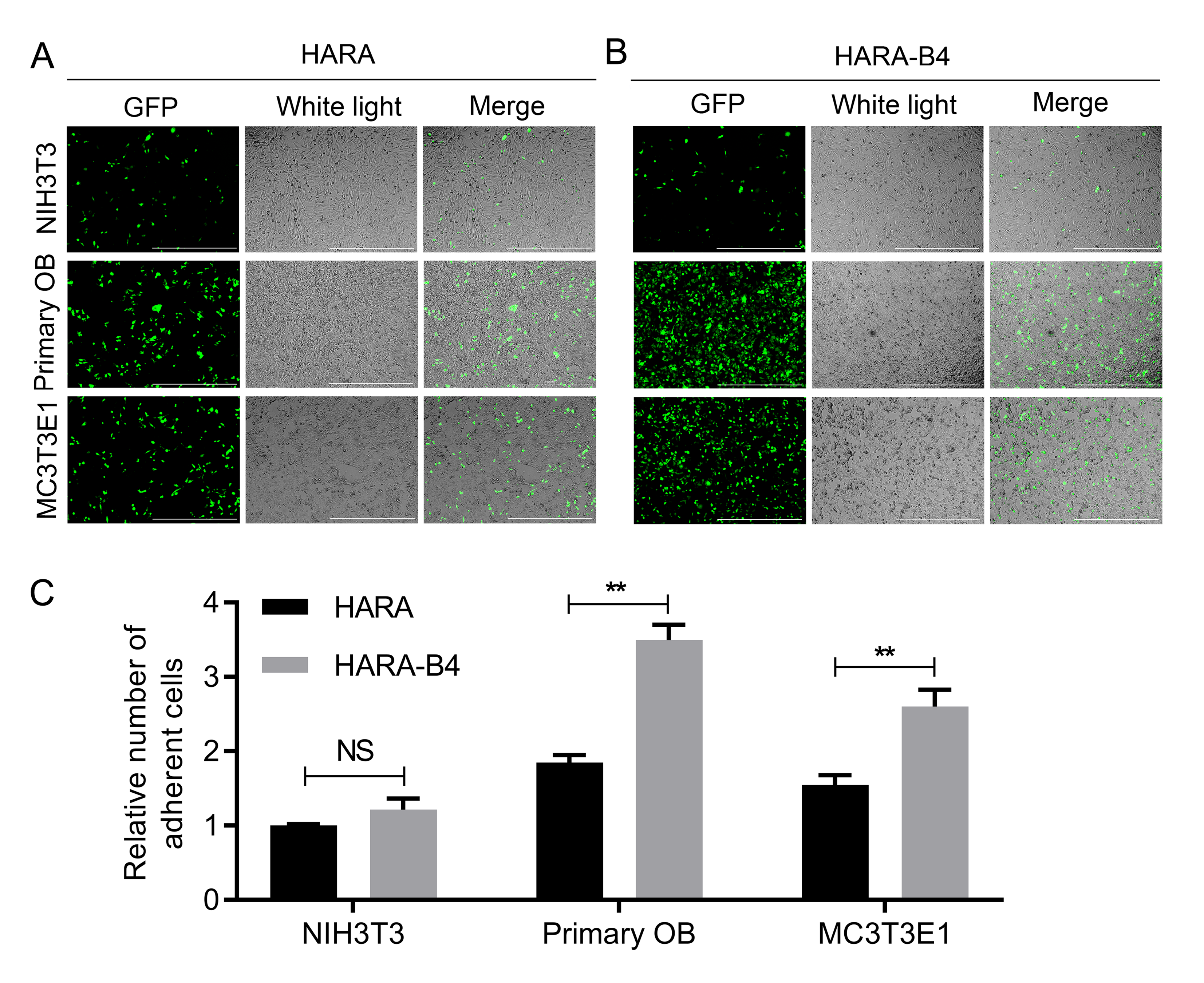

Supplement: Supplementary Figure 2 — HARA-B4 cells showed a stronger adhesive ability to osteocytes than did HARA cells HARA and HARA-B4 cells that had been transfected with the GFP plasmid were co-cultured with NIH3T3, Primary OB or MC3T3E1 cells. (A–C) The adhesive ability of HARA and HARA-B4 cells was measured. NS: not significant. P < 0.01, ** vs. HARA cells. [file Image_2.tif]
